# A Case of 5-Fluorouracil-Induced Hyperammonemia and Lactic Acidosis Successfully Treated With Continuous Hemodiafiltration Dialysis

**DOI:** 10.7759/cureus.72536

**Published:** 2024-10-28

**Authors:** Taro Asano, Narumi Yamada, Chikaaki Nakamichi, Takayuki Miyoshi, Hiroaki Takeshita

**Affiliations:** 1 Department of Emergency Medicine, National Organization Nagasaki Medical Center, Nagasaki, JPN; 2 Department of Surgery, National Organization Nagasaki Medical Center, Nagasaki, JPN

**Keywords:** consciousness, fluorouracil, hemodiafiltration, hyperammonemia, lactic acidosis, tomography

## Abstract

A 51-year-old male patient with stage IVc upper rectal cancer received treatment with aflibercept beta and folinic acid, fluorouracil, and irinotecan. Two days following treatment, he presented with an altered mental status. Head computed tomography showed no abnormalities, but blood tests revealed hyperammonemia and lactic acidosis. As these findings were thought to be side effects of 5-fluorouracil (5-FU), the patient underwent continuous hemodiafiltration. Subsequent to this intervention, the hyperammonemia, lactic acidosis, and impaired consciousness improved, and the patient was discharged on hospitalization day 10. In cases of impaired consciousness during 5-FU administration, in addition to the usual evaluation of the cause of impaired consciousness, ammonia and lactate levels should be measured, and branched-chain amino acids should be administered if impaired consciousness due to hyperammonemia is strongly suspected, and the patient's general condition is stable. If the disturbance of consciousness is prolonged or the patient's general condition becomes unstable, it is advisable to consider initiating blood purification. Additionally, patients with lactic acidosis or unstable general conditions should be considered for blood purification using continuous hemodiafiltration. Therapeutic interventions may lead to neurological improvement even in severe cases, highlighting the importance of prompt and targeted management approaches.

## Introduction

The pyrimidine metabolic antagonist antineoplastic agent 5-fluorouracil (5-FU) exerts antitumor effects by inhibiting DNA synthesis and is utilized to treat advanced, recurrent colorectal cancer and other malignancies. It has the potential to induce hyperammonemia accompanied by impaired consciousness as an adverse effect, although the incidence rate remains unclear. Additionally, lactic acidosis has been reported as a rare complication. Although 5-FU is a commonly used anticancer drug, there are few case reports of successful treatment due to hyperammonemia and lactic acidosis [1-5]. When indicated for treatment, therapeutic intervention may provide neurological improvement even in cases of advanced cancer and severe disease. We present the case of a patient who developed hyperammonemia and lactic acidosis after 5-FU administration and was successfully treated with continuous hemodiafiltration (CHDF).

## Case presentation

A 51-year-old male patient presented to the emergency department complaining of general malaise, anorexia, frequent vomiting, and impaired consciousness. He had a medical history of upper rectal cancer and was undergoing chemotherapy at our hospital. Two days before admission, he had completed the eighth course of chemotherapy with folinic acid, fluorouracil, and irinotecan (FOLFIRI; irinotecan: 150 mg/m2 on day one, l-leucovorin: 200 mg/m2 on day one, 5-FU: 400 mg/m2 on day one, 5-FU: 2,400 mg/m2 continuous IV infusion for 46 hours on days one and two) in combination with aflibercept beta (4 mg/kg). The patient had a history of chronic kidney disease. He was taking furosemide, oxycodone, and nicardipine, but was not on any medications that could potentially cause drug-induced lactic acidosis. He had lost approximately 20 kg in six months but remained independent in activities of daily living.

Upon arrival at the Emergency Department, the patient’s vital signs were as follows: Glasgow Coma Scale, 3/15 (E1V1M1); respiratory rate, 30 beats/minute; oxygen saturation (SpO2), 99% (on room air); blood pressure (BP), 134/88 mmHg; heart rate, 150 beats/minute; and body temperature, 36.7°C. A physical examination revealed normal breathing sounds without additional lung sounds. His heart sounds were normal, without any heart murmurs. The abdomen was non-distended and soft, and bilateral pedal edema was observed. There were no signs of sweating or cyanosis. A neurological examination revealed bilateral pupils measuring 5.0 mm with brisk light reflexes. Paralysis of the extremities was not observed. Echocardiography revealed preserved left ventricular systolic function, but the left ventricular cavity and inferior vena cava were collapsed, suggesting intravascular dehydration. The laboratory findings showed elevated ammonia levels of 1631 μg/dL, pH of 6.988, partial pressure of carbon dioxide of 18.6 mmHg, partial pressure of oxygen of 52.1 mmHg, bicarbonate level of 4.2 mmol/L, base excess of -25.9 mmol/L, and lactate level of >270 mg/dL. The findings were indicative of metabolic acidosis and lactic acidosis. Renal function tests showed a blood urea nitrogen level of 56.7 mg/dL and a creatinine level of 3.36 mg/dL, leading to a diagnosis of acute kidney injury. Additionally, the patient presented with hyperkalemia with a potassium level of 6.8 mEq/L (Table 1). 

**Table 1:** Laboratory findings APTT, activated partial thromboplastin time; WBC, white blood cell; RBC, red blood cell; Hb, hemoglobin; Hct, hematocrit; MCV, mean corpuscular volume; PT-INR, prothrombin time-international normalized ratio; FBG, fasting blood glucose; FDP, fibrin and fibrinogen degradation product; TP, total protein; Alb, albumin; T-bil, total bilirubin; AST, aspartate aminotransferase; ALT, alanine aminotransferase; CK, creatine kinase; BUN, blood urea nitrogen; CRP, C-reactive protein; NH3, ammonia; PCT, procalcitonin; Na, sodium; K: potassium; Cl, chlorine; pH, potential of hydrogen; PCO2, partial pressure of carbon dioxide; PO2, partial pressure of oxygen; HCO3, bicarbonate; BE, base excess; Glc, glucose; Lac, lactate

Test	Result	Reference range	Units
WBC	26.8	3.3~8.6	10^3/μL
RBC	3.93	4.35~5.55	10^3/μL
Hb	11.4	13.7~16.8	g/dL
Hct	39.5	40.7~50.1	%
MCV	100.5	83.6~98.2	fl
Plt	522	15.8~34.8	10^3/μL
PT	51	70~130	%
PT-INR	1.44		
APTT	22.4	26.0~38.0	sec
Fbg	730	200~400	mg/dL
FDP	21.9	<5	μg/mL
TP	7.5	6.6~8.1	g/dL
Alb	3.8	4.1~5.1	g/dL
Glb	3.7	2.2~3.4	g/dL
T-bil	0.5	0.4~1.5	mg/dL
AST	22	13~30	U/L
ALT	14	10~42	U/L
ALP	148	38~113	U/L
LD	259	124~222	U/L
G-GTP	62	13~64	U/L
CK	55	59~248	U/L
BUN	56.7	8.0~20.0	mg/dL
Crea	3.36	0.65~1.07	mg/dL
CRP	2.087	<0.14	mg/dL
NH3	1631	12~66	μg/dL
PCT	0.83	<0.05	ng/mL
Na	141	138~145	mEq/L
K	6.8	3.6~4.8	mEq/L
Cl	99	101~108	mEq/L
pH	6.988	7.350~7.450	
PCO2	18.6	32.0~48.0	mmHg
PO2	52.1	83~108	mmHg
HCO3	4.2	22.0~26.0	mmol/L
BE	-25.9	-2~+2	mmol/L
Lac	>270	4.5~14.4	mg/dL
Glc	207	70~105	mg/dL

Head computed tomography (CT) showed no evidence of hemorrhagic lesions or brain metastases, while abdominal CT showed no evidence of liver metastases or hydronephrosis after bilateral ureteral stenting. There were no abnormalities in the spleen, pancreas, adrenal gland, or intestinal tract. Consequently, the patient was diagnosed with hyperammonemia and lactic acidosis caused by 5-FU. Despite the patient being in the terminal stage of rectal cancer and there being difficulty determining indications for intensive care, treatment was initiated because acute blood purification can improve these conditions. CHDF was performed because of his hemodynamic instability. The CHDF settings were as follows: hemofilter, cellulose triacetate; membrane area, 2.1 m2. The blood flow rate was set at 100 mL/minute, the filtration flow rate at 1000 mL/hour, the dialysate flow rate at 500 mL/hour, and the replacement fluid flow rate at 500 mL/hour. On the day after admission and commencement of CHDF, the serum ammonia level was 26 μg/dL, and the lactate level was 24 mg/dL. Additionally, a urine output of approximately 100 mL/hour was achieved. On the third day, his level of consciousness had improved, leading to his successful weaning from CHDF. On the fourth day, the patient was extubated, and mechanical ventilation was discontinued. He demonstrated oral intake ability and was discharged home on the 10th day.

## Discussion

**Figure 1:**
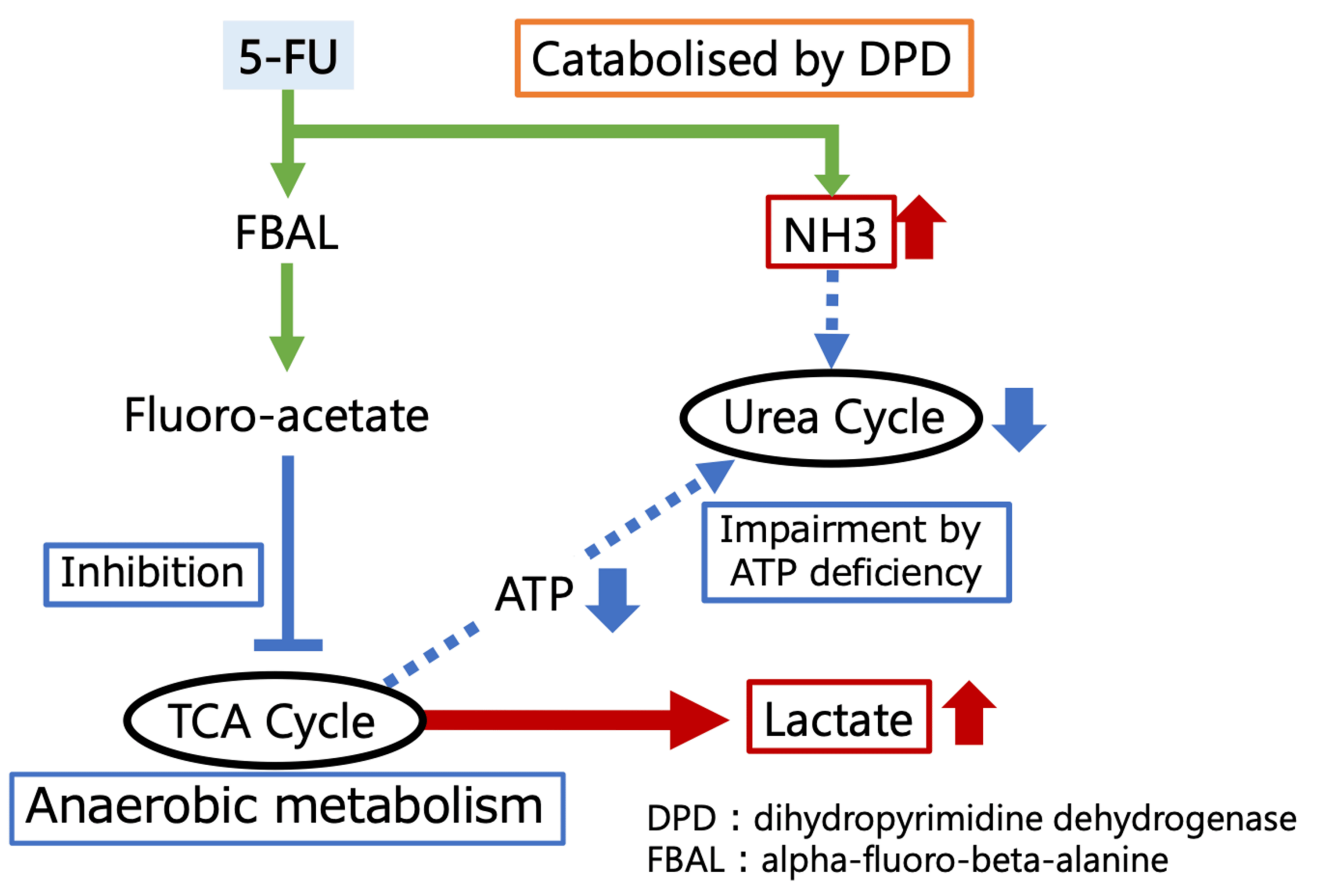
The mechanism by which 5-fluorouracil (5-FU) induces hyperammonemia and lactic acidosis This figure demonstrates the mechanism by which 5-FU metabolism in the liver induces hyperammonemia and lactic acidosis, primarily through the inhibition of the tricarboxylic acid (TCA) cycle and the disruption of ammonia (NH3) metabolism. Image Credit: Author Taro Asano

Herein, we report the case of a patient who developed hyperammonemia and lactic acidosis after 5-FU administration. In general, the risk factors for developing hyperammonemia with 5-FU administration include renal dysfunction, constipation, weight loss, and malnutrition [6]. However, there are no reported cases of risk factors for the development of lactic acidosis. The mechanism underlying the hyperammonemia and lactic acidosis induced by 5-FU involves its hepatic metabolism by dihydropyrimidine dehydrogenase, resulting in the formation of alpha-fluoro-beta-alanine and ammonia, which is further metabolized to fluoro-acetate, inhibiting the tricarboxylic acid (TCA) cycle. When the TCA cycle is inhibited, anaerobic metabolism increases, and adenosine triphosphate (ATP) production decreases, consequently elevating lactic acid levels and precipitating lactic acidosis. ATP depletion and reduced urea cycle activity can lead to ammonia retention and encephalopathy (Figure 1).

Ammonia metabolism occurs mainly in the liver; however, the skeletal muscle is also responsible for ammonia metabolism, suggesting that sarcopenia may be a predictive marker for the development of hyperammonemia [7]. Although many reports have shown that treating hyperammonemia with branched-chain amino acid preparations improves the condition, some patients require hemodialysis [4,8,9]. The removal rate of 5-FU by dialysis was reported to be 79.5%; therefore, hemodialysis is considered effective [10]. Regarding the survival and neurological prognoses of hyperammonemia, a national survey in France reported a mortality rate of 17% and a complete neurological recovery rate of 70% [11]. During the period from 1993 to 2022, five cases of lactic acidosis alongside hyperammonemia have been reported in PubMed and Ichushi (a medical journal database in Japan), with four of them requiring hemodialysis. All patients showed neurological improvement or were discharged from the hospital (Table 2) [1-5]. 

**Table 2:** The five cases of lactic acidosis alongside hyperammonemia reported in PubMed and Ichushi, during the period from 1993 to 2022 BCAA, branched-chain amino acid; CHDF, continuous hemodiafiltration; GCS15: A Glasgow Coma Scale (GCS) score of 15; FOLFIRI: folinic acid, fluorouracil, and irinotecan hydrochloride; mFOLFOX6: modified FOLFOX-6, a combination of folinic acid, fluorouracil, and oxaliplatin; FP: chemotherapy protocol combining 5-fluorouracil (5-FU) and cisplatin (CDDP); AFL: aflibercept beta

Author	Year	Age	Sex	Origin	Stage	Chemotherapy	NH3 (μg/dL)	Lactate(mg/dL)	Treatment	Outcome
Hara et al. [1]	2019	68	Female	Colorectum	Ⅳc	FOLFIRI	203	168.5	CHDF	GCS15
Sato et al. [2]	2019	83	Male	Rectum	Ⅳc	mFOLFOX6	1769.2	225.2	CHDF	GCS15
Kobayashi et al. [3]	2020	65	Male	Esophagus	N/A	FP	1325	114.4	CHDF	Discharged on 17th day
Fukuda et al. [4]	2020	71	Male	Rectum	Ⅲ	mFOLFOX6	1163	159.5	BCAA	Discharged on 7th day
Imai et al. [5]	2021	67	Male	Stomach	Ⅳ	mFOLFOX6	645	144.1	CHDF	GCS15
Present case	2022	51	Male	Rectum	Ⅳc	AFL＋FOLFILI	1631	＞270	CHDF	Discharged on 10th day

In cases of impaired consciousness during 5-FU administration, in addition to the usual examination of the cause of impaired consciousness, ammonia and lactate levels should be measured, and branched-chain amino acids should be administered if impaired consciousness due to hyperammonemia is strongly suspected, and the patient's general condition is stable. Prolonged impairment or unstable general conditions warrant consideration for blood purification. Additionally, patients with lactic acidosis or unstable general conditions should be considered for blood purification using CHDF. In terms of treatment indications, neurological improvement may be achieved with therapeutic intervention, even in severe cases. The patient's chronic kidney disease and significant weight loss over the past six months likely contributed to metabolic disturbances and increased sensitivity to the toxic effects of 5-FU. In cases involving patients with similar risk profiles, these factors should be carefully considered. As with the cases reported by Sato et al. and Kobayashi et al., this patient developed lactic acidosis in addition to hyperammonemia and exhibited hemodynamic instability, necessitating CHDF for the removal of ammonia, lactate, and 5-FU [2,3]. Although few cases have been reported to date, CHDF appears to be a potentially effective option for managing these complications, particularly in patients with risk factors such as renal impairment.

## Conclusions

We report a case of hyperammonemia and lactic acidosis following postoperative administration of 5-FU in a patient undergoing treatment for rectal cancer. The patient achieved successful recovery with CHDF. Although the simultaneous occurrence of hyperammonemia and lactic acidosis is uncommon, this case highlights the critical importance of early detection and prompt management in optimizing patient outcomes. In particular, for patients with predisposing factors such as renal impairment, it is advisable to monitor ammonia and lactate levels during 5-FU therapy. In cases where 5-FU-induced hyperammonemia and lactic acidosis develop, CHDF may serve as an effective therapeutic option when conventional treatments fail or in the presence of hemodynamic instability.
